# Modulation of natural killer cell exhaustion in the lungs: the key components from lung microenvironment and lung tumor microenvironment

**DOI:** 10.3389/fimmu.2023.1286986

**Published:** 2023-11-06

**Authors:** Hongxia Zhang, Jian Wang, Fengqi Li

**Affiliations:** ^1^ Institute of Health and Medicine, Hefei Comprehensive National Science Center, Hefei, Anhui, China; ^2^ Department of Neurology, The First Affiliated Hospital of University of Science and Technology of China (USTC), Division of Life Sciences and Medicine, University of Science and Technology of China, Hefei, Anhui, China

**Keywords:** natural killer cells, hypofunction, exhaustion, lung microenvironment, lung tumor microenvironment

## Abstract

Lung cancer is the leading cause of tumor-induced death worldwide and remains a primary global health concern. In homeostasis, due to its unique structure and physiological function, the lung microenvironment is in a state of immune tolerance and suppression, which is beneficial to tumor development and metastasis. The lung tumor microenvironment is a more complex system that further enhances the immunosuppressive features in the lungs. NK cells are abundantly located in the lungs and play crucial roles in lung tumor surveillance and antitumor immunity. However, the immunosuppressive microenvironment promotes significant challenges to NK cell features, leading to their hypofunction, exhaustion, and compromised antitumor activity. Thus, understanding the complex interactions among the lung microenvironment, lung tumor microenvironment, and NK cell exhaustion is critical for the development of effective cancer immunotherapeutic strategies. The present review will discuss NK cell hypofunction and exhaustion within the lung microenvironment and lung tumor microenvironment, focusing on lung tissue-specific factors, including key cytokines and unique environmental components, that modulate NK cell activation and function. Understanding the functional mechanisms of key factors would help to design strategies to reverse NK cell exhaustion and restore their antitumor function within the lung tumor microenvironment.

## Introduction

Lung cancer is a disease with very high morbidity and mortality worldwide in both sexes (1). In addition, the lung is the primary site for disseminated tumor seeding (2). The continuous interaction on the open surface and rich capillary network of the lungs brings a higher chance of making tumors “seed” into the lungs, and the immunosuppressive microenvironment of the lungs provides “soil” for tumor growth, so the lung becomes a high incidence site of tumors *in situ* and metastases. For example, breast cancer with very high morbidity in the world has lung metastasis in 60-70% of relapsed patients (3). Natural killer (NK) cells are an important innate lymphocyte population that are abundant in the lungs and play critical roles in the antitumor immune response. NK cells mediate antitumor effects by producing effector cytokines or exerting direct cytotoxic activity (4). However, NK cells in the lungs display inactivated and hypofunctional features (5). When the tumor microenvironment is formed in the lungs, NK cells are getting exhausted (6, 7). This review summarized the roles of the main lung-specific components that can modulate NK cell hypofunction and exhaustion in both the lung microenvironment and lung tumor microenvironment. Furthermore, future studies on promising antitumor strategies will also be discussed.

## The lung is a unique, complex, and delicate organ

As the main organ for oxygen and carbon dioxide exchange, the lung is essential for mammalian survival. The surface area of the lower respiratory tract is approximately one hundred square meters in a healthy adult. Furthermore, the alveoli are the terminal units of the respiratory tract tree, and they are highly vascularized and have thin-walled architecture. The surface area of capillaries encompassing the alveoli is approximately 140 square meters. Moreover, the basement membrane of the alveolocapillary, which is the interface of alveolar epithelium and capillary endothelium, is only approximately 0.3 μm in thickness. To meet the active metabolic demands of the body, the distal airway of a healthy human adult needs to filter approximately ten thousand liters of air every day, and the pulmonary capillaries need to handle approximately 5 liters of blood every minute. Meanwhile, the large thin tenuous lung barrier is persistently exposed to different kinds of allergens, particles, and pathogens inhaled from the air and carried from the blood (8). Thus, the unique physiological functions and structures determine that the lung is a complex and delicate organ.

The physiological functions and the complex environment of the lungs require that the immune response in the lungs must be fast and efficient but tightly controlled to protect the tenuous lung barrier from excessive immune responses and inflammation and maintain immune homeostasis (9–11). The lung possesses a unique immune regulatory network that consists of structural cells and resident immune cells. All these cells are involved in the mounting of the lung-specific microenvironment (12).

## Lung immune cells in homeostasis and disease

The lung has a unique set of immune cells, including innate and adaptive immune cells that are located within the lung niche and mediate the lung-specific immune response in homeostasis and disease (12). In the lungs, the particles, allergens, and pathogens from the inhaled air are removed mainly by lung-resident macrophages and dendritic cells (DC). Alveolar macrophages (AMs) are found in the alveoli, where they are composed of 90-95% of immune cells in the steady state in mice (13). Another type of lung-resident macrophage is interstitial macrophages (IMs), which are divided into two subsets. Lyve1^hi^ IMs are closely located with blood vessels and play a crucial role in maintaining blood vessel integrity and anti-fibrotic activity. Lyve1^lo^ IMs are situated close to lung nerve bundles and display higher antigen-presentation capacities (14). Lung resident DCs are mainly located at the basolateral side of the epithelium and are important for initiating appropriate immune responses to inhaled antigens (13). In addition, different types of lymphocytes/lymphoid cells are found in the lungs at steady state, including T cells, B cells, NK cells, and innate lymphoid cells (ILCs). Increased evidence has shown that lung-resident memory T and B cells, approximately 80% of lymphocytes in total in mouse lungs, play an important role in protecting against respiratory reinfection (15). ILCs also mediate a protective immune response from pathogens and promote tissue repair and homeostasis after infections (16), although the total lung ILC population is approximately 20–30 thousand cells in naive mouse lungs and only 0.1% of all CD45^+^ cells in human lungs (17). Furthermore, as a critical part of lymphocytes, lung NK cells are crucial effector lymphocyte populations in antitumor and anti-infection immune responses (18, 19). In this review, we will focus on lung NK cells and discuss their phenotype and function in the lung microenvironment.

## The lung microenvironment shapes the specificity of lung NK cells in homeostasis

As important innate lymphocytes, NK cells are widely distributed in lymphoid and non-lymphoid tissues and play a crucial role in immune surveillance (20, 21). The lung is an important barrier of the body and is full of interactions between immune cells and foreign pathogens. NK cells are abundant in the lungs of both humans and mice (22, 23), indicating the significance of lung NK cells. There are high percentages of NK cells among lymphocytes in the lungs, approximately 10% in mice and 10-20% in humans, compared to other lymphoid and non-lymphoid organs, such as lymph nodes, bone marrow, spleen, blood, and liver (24–26), and the cytokine interleukin (IL)-15 derived from epithelial cells and alveolar macrophages might contribute to the high percentages of NK cells in the lung microenvironment (27, 28).

In normal lungs, NK cells are located in the lung interstitium. NK cells infiltrate into the alveoli and are observed in the bronchoalveolar fluid (BAL) during respiratory infection and in lung inflammatory diseases (29). In 1980, the antitumor function of NK cells was proven by three independent groups at almost the same time (30–32). Talmadge et al. found that NK cells have an important function in controlling tumor growth and metastasis in an NK cell-deficient mouse model (30). Karre et al. reported that NK cell-deficient mice display low *in vivo* resistance to syngeneic leukemias (31). Roder et al. observed that patients with dysfunctional NK cells have a profound defect in their ability to spontaneously lyse various tumor cells *in vitro* (32). Since then, an increasing number of studies have confirmed that NK cells have antitumor effects in multiple different tumors (33). In lung cancer patients, a correlation between tumor-infiltrating NK cells and a better prognosis has been observed (34, 35). In the *Kras*-driven spontaneous lung cancer and lung tumor implantation mouse model, NK cells showed antitumor effects. In both studies, mice lacking NK cells showed a higher tumor burden (36, 37). In addition to their antitumor function, NK cells are involved in immune responses to lung infections. In humans, increased respiratory viral infections are associated with dysfunction of NK cells (38, 39). However, studies with mouse models show that lung NK cells have a protective role in controlling viral and bacterial burden after infection (40). On the other hand, NK cells also contribute to inflammation-mediated damage and exacerbate the pathology of infection (41). The different functions of NK cells are driven by the local lung tissue microenvironment.

Lung NK cells display unique features compared to NK cells in other tissues: i) Lung NK cells maintain a more mature phenotype. In human lungs, NK cells are found in the parenchyma, and most of them display a fully differentiated CD56^dim^CD16^+^ phenotype. However, NK cells in the liver and other lymphoid organs display a CD56^bright^CD16^–^ phenotype (19, 42). Similarly, in mouse lungs, most NK cells exhibit a mature CD27^-^CD11b^+^ phenotype (24); ii) The majority of lung NK cells are not tissue-resident. In both humans and mice, only a few lung NK cells express CD49a, which is a tissue-resident NK marker, indicating that lung NK cells mainly circulate between blood and the lungs (5, 19); iii) Lung NK cells display an inhibitory phenotype. In mice, compared to NK cells isolated from the spleen and bone marrow, lung NK cells express lower levels of activation-related molecules, such as CD69, NKp46, and NKG2D, and higher levels of inhibitory receptors CD94 and NKG2A (24). In humans, lung NK cells also express killer Ig-like receptors (KIRs), indicating that lung NK cells are quiescent in a steady state (5); iv) Lung NK cells are hypofunctional. In the 1980s, human NK cells were found to be functionally important in the lungs (42). Lung NK cells showed impaired cytotoxic abilities compared to NK cells from peripheral blood. The main structural cells (lung epithelial cells) and resident immune cells (alveolar macrophages) in the lungs are involved in the process of NK cell hypofunction (43–46). The key environmental components in the lungs, such as prostaglandin E2 (PGE2), TGF-β, and pulmonary surfactant, are the main mediators and are responsible for impaired NK cell functions (46). Recently, further studies have shown that PGE2 produced by lung fibroblasts is the main mechanism to explain the inhibition of NK cell functions (2). However, the inhibitory phenotype of NK cells in homeostasis could be reversed after respiratory viral or bacterial infection, and NK cells have the potential to be activated rapidly and involved in immunopathology in an inflammatory lung microenvironment (24). Overall, the particular phenotype and special features of lung NK cells under physiological conditions are shaped by the unique lung microenvironment and are beneficial for avoiding unnecessary inflammation and maintaining immune homeostasis in the lungs.

## The lung tumor microenvironment causes exhaustion of lung NK cells

NK cells play critical roles in tumor surveillance and antitumor immunity in the circulating system and multiple organs (47). NK cells can be activated directly to eliminate target cells without priming (20). NK cells perform their effector function in several different ways. After activation, NK cells can directly kill transformed cells or tumor cells by producing perforin and granzyme B (48, 49) or by inducing target cell death through tumor necrosis factor (TNF)-α, Fas ligand (FasL), and TNF-related apoptosis-inducing ligand (TRAIL) (50–52). In addition, NK cells can eliminate transformed cells or tumor cells through antibody-dependent cell-mediated cytotoxicity (ADCC) in the presence of antibodies (53). Furthermore, NK cells can secrete different cytokines and chemokines that subsequently promote other immune cells to exert antitumor functions (54).

However, in the condition of lung cancer, NK cells display an exhaustion status with an altered phenotype and impaired effector function. In mice, NK cells could prevent lung tumor initiation in a *Kras*-driven lung cancer model. However, once the tumor enters the promotion and progression stage, NK cells exhibit an exhausted phenotype with low cytotoxicity and viability (36). Similarly, intratumor NK cells show an exhausted phenotype, lower IFN-γ production, and impaired cytotoxic function in patients with non-small cell lung carcinoma (NSCLC) (7, 55–57). Most recently, Tang et al. analyzed human NK cells from 716 patients across 24 tumor types by integrative single-cell sequencing and found that NK cells display tumor-type preferences and are associated with both intrinsic organ properties and factors from the tumor microenvironment. The proportions of CD56^dim^CD16^hi^ NK cells are significantly decreased in lung cancer compared with noncancer lungs. Further transcriptomic feature analysis in this study has shown that a subset of tumor-associated NK cells is enriched in tumors and displays exhaustion features and impaired antitumor functions in multiple cancers, including lung cancer (58). Thus, understanding the mechanisms and key factors that promote NK cell exhaustion in the lung tumor microenvironment would provide new directions for antitumor strategies.

## Unique components of the lung microenvironment and lung tumor microenvironment promote lung NK cell exhaustion

**Figure 1:**
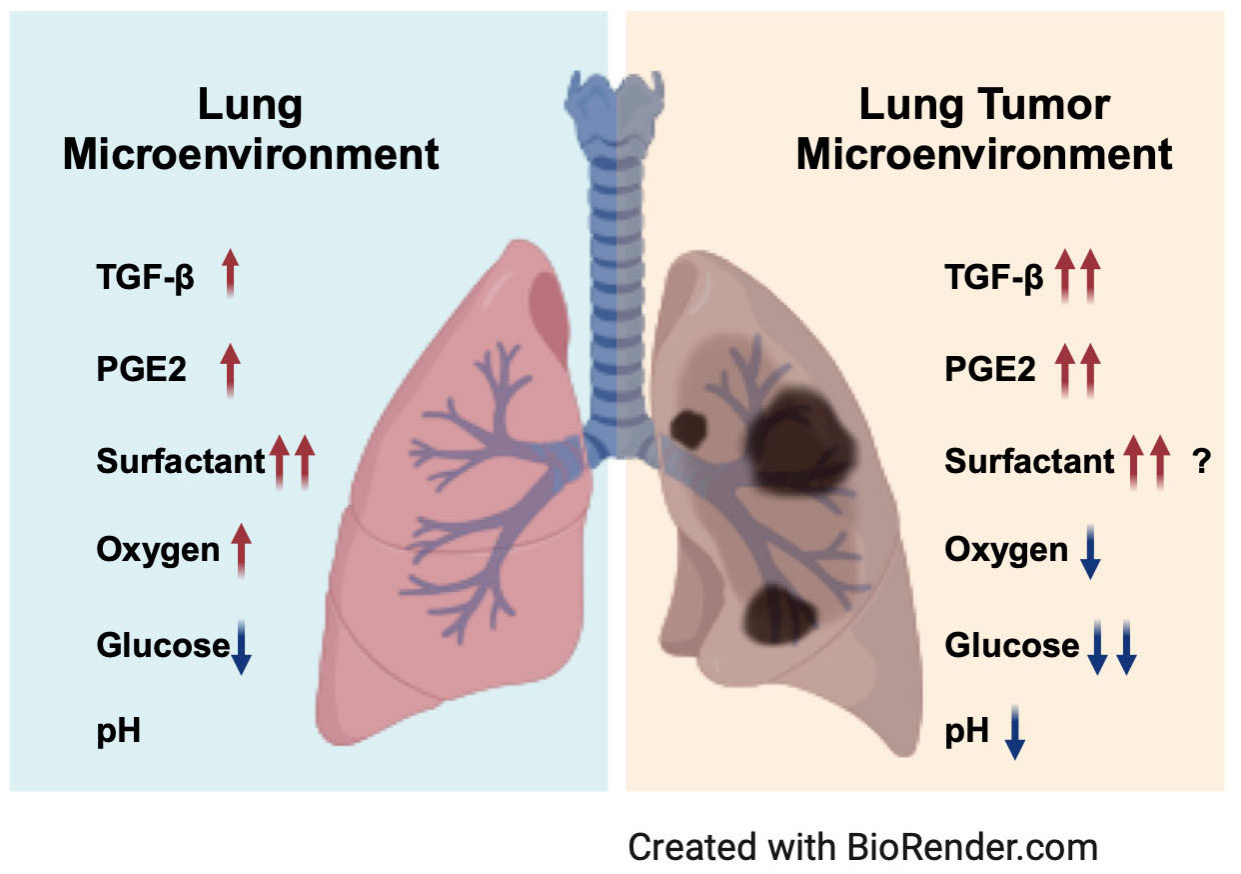
Key components from the lung microenvironment and lung tumor microenvironment. Abundant TGF-β, PGE2 and surfactant in the lung microenvironment are crucial for lung homeostasis. High oxygen and low glucose are special features of the lung microenvironment. When tumors grow in the lungs, more TGF-β and PGE2 are produced, and the physical and chemical environment changes to low oxygen, lower glucose, and low pH. Created with BioRender.com.

**Figure 2:**
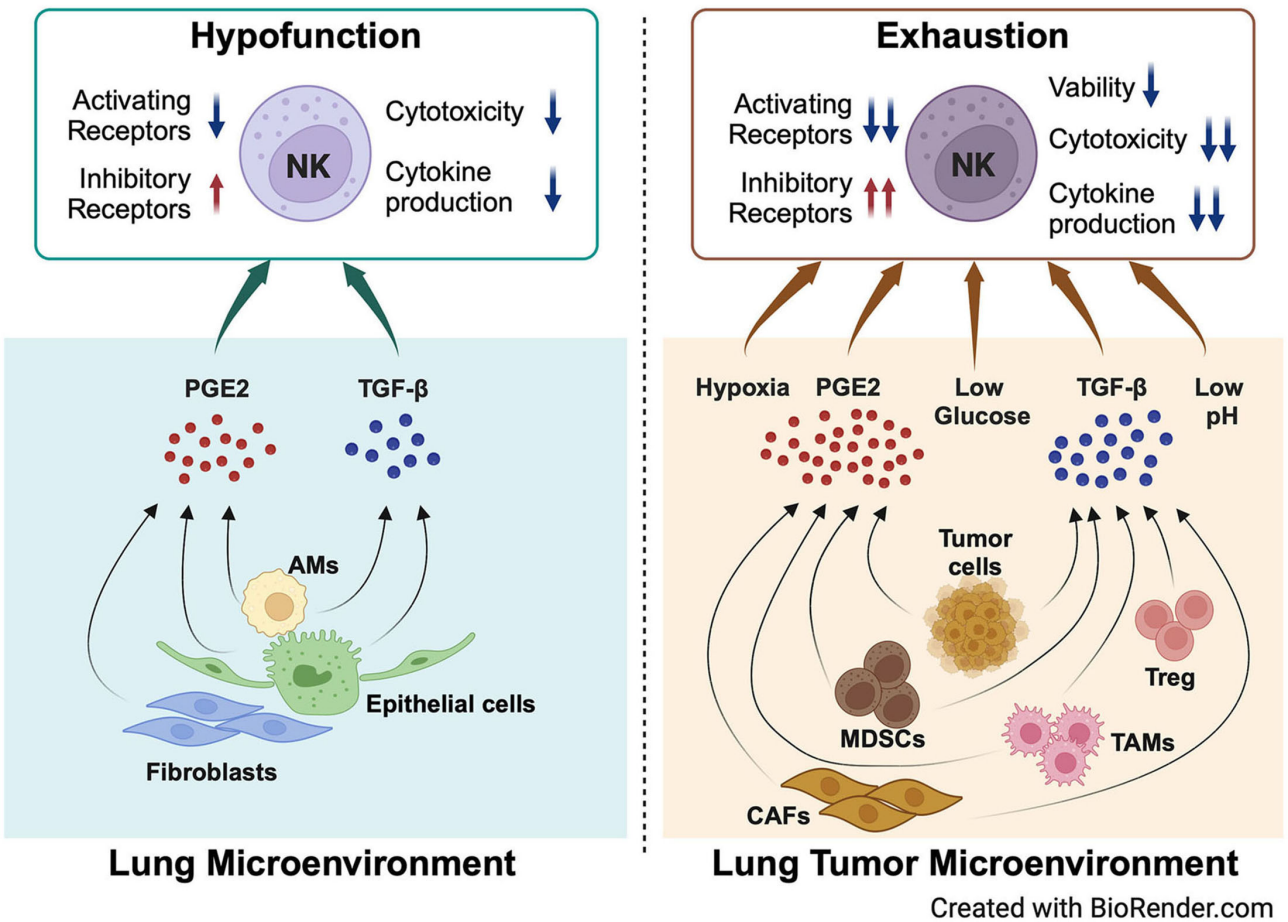
NK cell hypofunction and exhaustion are modulated by key components of the lung microenvironment and lung tumor microenvironment. PGE2 and TGF-β secreted by lung epithelial cells, AMs, and fibroblasts in the lung microenvironment modulate the hypofunction of lung NK cells in homeostasis. When tumors grow in the lungs, more PGE2 and TGF-β are secreted by multiple cell types, including tumor cells, MDSCs, TAMs, CAFs, and Tregs. The physical and chemical environment changes to hypoxia, lower glucose, and low pH. All these factors promote NK cell exhaustion in the lung tumor microenvironment. Created with BioRender.com.

Based on the special structure and composition of structural cells and immune cells, the lungs have many unique intrinsic features. For example, the expression of PGE2 in the lungs is relatively higher than that in other tissues (2). Furthermore, the expression of TGF-β in the lungs is high during lung development (59). There are also other unique chemical conditions, such as high levels of oxygen (60, 61), low levels of glucose (62–64), and abundant surfactant protein and lipids (65) in the alveoli (**Figure 1**). All these special components are involved in shaping the inactivation and hypofunctional status of NK cells. When tumors grow in the lungs, there are more immunosuppressive cells infiltrating the lung tumor microenvironment, such as myeloid-derived suppressor cells (MDSCs), tumor-associated macrophages (TAMs), and regulatory T cells (Tregs) (66). These types of cells produce more PGE2 and TGF-β (67, 68) (**Figure 2**). Moreover, the physical and chemical environment is changing to hypoxia, low pH, and lower glucose (69–71) (**Figures 1**, **2**). All these mediators further promote NK cells changing to exhaustion status with altered phenotype and impaired function.

### TGF-β

TGF-β is a critical immunosuppressive cytokine of the lungs in a steady state and plays critical roles in driving AMs proliferation and differentiation and maintaining lung immune homeostasis (72). TGF-β is produced by multiple cell types in the normal lung microenvironment (12). In 1984, Robinson and colleagues found that NK cells isolated from lung tissue display impaired cytotoxic function (42). Later, several studies have proven that exogenous TGF-β could inhibit the IFN-γ production and activity of human NK cells *in vitro*, indicating the role of TGF-β in shaping special phenotypes and impaired function of lung NK cells (73–75).

As a biomarker of tumors and an extensive inducer of the tumor microenvironment, TGF-β is produced by different types of cells and plays multiple roles in tumor progression. Lung tumor cells themselves can secrete large amounts of TGF-β (76). TGF-β can induce tumor formation by promoting the process of epithelial-mesenchymal transformation (EMT) (77, 78). TGF-β also facilitates the transformation of fibroblasts into cancer-associated fibroblasts (CAFs), and CAFs can be the main source of TGF-β to further elevate the levels of TGF-β in the lung tumor microenvironment (79). TGF-β promotes CD4^+^ T cells to differentiate into Tregs and macrophages to polarize to M2 cells (80). Furthermore, with the formation of a tumor microenvironment, large numbers of TAMs, MDSCs, and Tregs appear in lung tumors (66). These cells could also secrete TGF-β, which further promotes NK cell exhaustion (**Figure 2**). TGF-β promotes NK cell exhaustion in several different ways. First, TGF-β alters the receptor-ligand interaction between NK cells and tumor cells. In response to TGF-β, NK cells downregulate the activating receptors NKG2D, NKp30, and NKp80 (81) and upregulate the inhibitory receptors NKG2A and PD1 (82). On the other hand, TGF-β also inhibits the expression of NKG2D ligands in human lung cancer cells (67). Second, TGF-β changes the metabolic features of NK cells. In the *Kras*-driven lung cancer mouse model, TGF-β inhibits glycolysis in NK cells by regulating the production of FBP1, which further mediates the exhaustion of NK cells (36). Finally, TGF-β induces microRNAs to silence NK cells. In human lung cancer, high levels of TGF-β deplete DAP12 by inducing microRNA-183 expression (83). Overall, in exposure to gradually increased TGF-β in the lung tumor microenvironment, lung NK cells are precisely regulated step by step and change themselves from hypofunction to exhaustion.

### PGE2

PGE2 is a lipid compound generated from arachidonic acid via the catalysis of the enzyme cyclooxygenase (COX), including COX1 and COX2, and it plays multiple roles in regulating different stages of the immune response (68). In healthy lungs, PGE2 is mainly secreted by lung fibroblasts (2). The expression level of *Ptgs2*, encoding COX2, is much higher in lung fibroblasts than in lung epithelial cells, endothelial cells, lung CD45^+^ immune cells, and fibroblasts from other tissues, such as bone, heart, liver, spleen, and mammary gland, in mice (2). Similarly, high levels of *PTGS2* expression were also detected in human lung tissues compared to other tissues through analyses of microarray data (2, 84). Gong et al. proved that the high level of PGE2 secreted by lung fibroblasts is responsible for the unique lung microenvironment, and the unique lung microenvironment endows the immunosuppressive phenotype of myeloid cells and the dysfunction of DCs in the lungs, which ultimately inhibits the cytotoxicity of NK cells (2) (**Figure 2**). The lung is the most common site for tumor metastasis (2). Deletion of *Ptgs2* in lung fibroblasts or inhibition of the PGE2 receptor EP2 and EP4 could reverse the immunosuppression of resident myeloid cells and diminish lung metastasis in several breast cancer models, indicating that PGE2 is a critical factor in mediating premetastatic niche formation (2). As an important component of the premetastatic niche, neutrophils secrete the inflammatory cytokine IL-1β in the lungs, which facilitates lung fibroblasts to produce more PGE2 and promote the formation of the lung tumor microenvironment. Furthermore, TGF-β could increase the production of PGE2 by augmenting the expression of COX-2 through a noncanonical pathway (80).

In the tumor microenvironment, an increased amount of PGE2 is secreted by multiple cell types, including tumor cells, TAMs, CAFs, and MDSCs (76, 85–87). Increased evidence has shown that PGE2 can inhibit the antitumor activity of NK cells in lung cancer (88, 89). A high level of PGE2 could also induce immunosuppressive FOXP3^+^ Treg cells in lung cancer (90, 91). In PGE2-producing tumors, fewer NK cells are in the tumor, and they lose the ability to produce the cytokines CCL5 and CXCL1 (92). Moreover, PGE2 downregulates the expression of the activating receptors NKp46, NKp44, NKp30, and NKG2D by binding to the receptors EP2 and EP4 on NK cells (93), then promoting the amplification of cyclic AMP cascade protein kinase A signaling (94), and finally resulting in the exhaustion of NK cells (95, 96) (**Figure 2**). Thus, PGE2 plays an important role in inducing NK hypofunction in the lung microenvironment and NK exhaustion in the lung tumor microenvironment.

### Oxygen

Since the critical physiological function of the lungs is to mediate the exchange of oxygen and carbon dioxide, the respiratory tract is a unique niche with relatively high levels of oxygen (61), which has a direct influence on lung immunity (60). Oxygen is transported by red blood cells from alveoli to different organs. Under physiological conditions, due to oxygen being delivered according to the metabolic requirements of each tissue, different tissues have their own special ‘physioxia’ status. The oxygen levels in the atmosphere, so-called normoxia, are 21.1% (160 mmHg). The oxygen levels are approximately 19% (150 mmHg) in the trachea, approximately 14.5% (110 mmHg) in the alveoli, and 13.2% in arterial blood, which is relatively higher than other tissues (61). Cells from different organs can sense oxygen levels and change their metabolic and transcriptional features. Moreover, inflammation and oxygen levels are tightly linked, and hypoxia can cause inflammation (97). In the lung microenvironment, hypoxia could induce fundamental biological actions in lung structure cells and alveolar macrophages. The decline in oxygen levels in the local environment leads to the expression and stabilization of the transcription factor hypoxia-inducible factor (HIF), which plays a key role in lung diseases (98). HIF1α and HIF2α can be expressed by pulmonary artery smooth muscle cells (PASMCs), endothelial cells, and epithelial cells (98). Furthermore, recent studies have shown that the Von Hippel− Lindau (VHL) protein, which is a negative regulator of HIF, directly controls the terminal differentiation, self-renewal, and function of alveolar macrophages in homeostasis (99, 100). All these changes induced by hypoxia in the lung microenvironment might modulate other immune cells, including NK cells.

When a tumor appears in the lungs, the oxygen levels are completely changed due to the rapid growth of tumor cells. Hypoxia can promote the progression of tumors in multiple ways (101). The hypoxia-induced pathway plays important roles in promoting EMT, maintaining tumor stem cells, promoting angiogenesis, and driving the activation and expansion of immune-suppressive stromal cells (101). After the formation of a typical tumor microenvironment, hypoxia could further induce NK cell exhaustion (102). HIF1α induced in NK cells suppresses the upregulation of activating receptors such as NKp46, NKp44, NKp30, and NKG2D in the presence of activating cytokines, and NK cells display impaired cytotoxic capacity to target cells (103). Hypoxia in NK cells induces lower cytokine production (104). Furthermore, NK cells from HIF1α KO mice increase cytokine secretion and display higher antitumor activity (105), further demonstrating the important role of HIF1α. Overall, the low levels of oxygen (hypoxia) in the lung tumor microenvironment could promote the phenotype and function of NK exhaustion, but the detailed mechanism is not clear, and more studies need to be done.

### Glucose

In homeostasis, the glucose concentration in the fluid line with lung epithelial cells is more than 10 times lower than that in blood. The glucose concentration in blood is maintained at approximately 4.0-5.5 mmol/L, which is the optimal level for normal brain functions (106). The liquid from the lower airways of healthy human donors contains only approximately 0.4 mmol/L glucose (106), which is critical to limit bacterial growth in mice and human lungs (62–64). Glucose levels in the alveoli increase when airway homeostasis is disrupted by inflammation and viral infection, and high glucose levels in blood are observed in patients with diabetes or acute hyperglycemia (64, 106). Low glucose levels in the alveoli are maintained against a concentration gradient by the highly activated glucose transporters Glut1 and Glut 10 (62). Elevated airway glucose concentrations may increase the risk of respiratory bacterial infection, particularly methicillin-resistant *Staphylococcus aureus* (MRSA) (64). High airway glucose concentrations might also exacerbate lung disease by inducing local inflammation. As the critical carbon source, the effect of glucose on inflammation and infection is higher than that of other nutrients (63). Interestingly, the glycolytic capacity of alveolar macrophages is relatively low compared to that of macrophages from other tissues (107), which might be explained by the low glucose levels in the alveolar niche. Normally, aerobic glycolysis is vital for T, B, and NK cell activation by supporting the biosynthetic demands of these cells (108). Thus, the inactivated state of lymphocytes might be partially associated with glucose shortage in the lung microenvironment.

Glucose restriction is also a biomarker of the lung tumor microenvironment (71). Cong et al. found that NK cells isolated from the lung tumor microenvironment have lower levels of glycolytic capacity accompanied by attenuated cytotoxic function and cytokine production (36). They also found that the increased expression of fructose-1,6-bisphosphatase (FBP1) inhibits glycolysis in NK cells from a lung cancer mouse model. These data indicate that tumor-driven glucose restriction inhibits glycolysis in NK cells and impairs their antitumor activity (109). On the other hand, the increased lactate concentration is also a biomarker of the TME, which is due to tumor cells primarily using glucose for glycolytic metabolism. Elevated lactate levels or low pH have been proven to prevent the cytotoxic activity of NK cells (110). Stimulation of NK cells by PMA/ionomycin in the presence of lactic acid could block their IFN-γ production (111). Overall, the correlation between glucose metabolism and NK cell exhaustion in the lung tumor microenvironment is largely unknown, and more research needs to be done.

### Surfactant

The alveoli are coated with a layer of surfactant, a highly surface-active phospholipid-rich material, which is essential for respiratory function in mammals (112). Pulmonary surfactant is a complex mixture of approximately 90% lipids and 8-10% protein, which is essential for reducing surface tension at the air-liquid interface of the terminal airways and preventing the alveolus collapse upon expiration (113, 114). Surfactant is produced in type II alveolar epithelial cells, where it is assembled into lamellar bodies, densely packed membranous acidic organelles (112, 115). Type II alveolar epithelial cells and alveolar macrophages are the main cells responsible for surfactant uptake and degradation (114). When inflammation or lung injury occurs, some of the surfactant components might leak into the blood. The appearance of surfactant proteins in plasma could be used as an early marker of lung injury (114).

#### Surfactant protein

There are four surfactant proteins (SP), SP-A, SP-B, SP-C, and SP-D in the alveoli. SP-B and SP-C are small and very hydrophobic proteins that are essential for forming a monolayer at the air-liquid interface and have lower surface tension. SP-B deficiency or gene mutation is associated with respiratory distress syndrome in neonates (116). Furthermore, gene mutation of SP-C induces pulmonary alveolar proteinosis-like disease in some cases (117). SP-A and SP-D are larger hydrophilic-related proteins of the collectin family, which have important roles in innate immunity and local immune modulation (118). SP-A and SP-D can directly bind respiratory pathogens, allergens, and particles through their collectin binding domain and mediate phagocytosis by lung-resident cells, which are important for pathogen clearance in the early stage of infection (119–121). Studies have shown that SP-A could increase AM alternative activation (122, 123), indicating that SP-A might be involved in mounting the anti-inflammatory microenvironment in the lungs. However, SP-A has also been reported to increase activated NK cell numbers and prevent lung cancer progression by promoting the polarization of M1 tumor-associated macrophages (124). SP-D was found to prevent lung cancer progression by downregulating epidermal growth factor signaling (125). Although the role of surfactant protein in lung cancer has been studied, its importance in NK cells remains largely unclear. More research is needed to investigate the direct role of surfactant protein in influencing NK cell functions.

#### Surfactant lipids

Approximately 75–80% of phospholipids are phosphatidylcholines. The most abundant component of surfactant lipids is saturated dipalmitoylphosphatidylcholine (DPPC), approximately 40% of the total lipids. DPPC contains two saturated palmitic chains (16:0/16:0 PC). In addition, other anionic phospholipids, such as phosphatidylglycerol (PG, approximately 7%), phosphatidylethanolamine (PE, approximately 3.7%), phosphatidylinositol and phosphatidylserine (5.4%), and sphingomyelin (6.8%), appear as components of surfactant (126, 127). Decreased levels of surfactant lipids have been described in smokers and COPD patients (128, 129). Surfactant phospholipids reduce alternative activation and proliferation of alveolar macrophages by decreasing the activation of PI3K-Akt-mTORC1 signaling (130, 131). Studies have shown that lipids purified from the BAL fluid of healthy donors inhibit NK cell cytotoxicity *in vitro* (132). However, different lipid components have distinct roles in NK cell activation. PG was found to suppress, while PE could augment, the cytotoxic function of NK cells (132). Recently, Gong et al. reported that lipid-laden lung mesenchymal cells facilitate breast cancer lung metastasis via metabolic reprogramming of tumor cells and NK cells. They found that lipid-laden mesenchymal cells could transport their lipids to tumor cells and NK cells via exosome-like vesicles, which leads to tumor cell proliferation and NK cell exhaustion (133). Despite the high abundance of surfactant lipids in the alveoli, their roles in inflammation and cancer are unknown, and more studies are necessary to understand the importance of each phospholipid in shaping the function of NK cells.

## Perspective

The effector function of NK cells is regulated by the fine balance between activating and inhibitory signals. Blockage of checkpoint receptors has displayed the potential to reverse NK cell exhaustion in tumors and boost their antitumor function. As a promising immunotherapy strategy for tumors, NK cell-based checkpoint blockade immunotherapy has displayed potential roles in improving current T-cell-based tumor immunotherapy. Recently, the therapeutic potential of many inhibitory receptors on NK cells, such as NGK2A, KIRs, TIM-3, TIGIT, CD96, and PD-1, has been proven by animal experiments or some clinical trials (102, 134). However, NK cell-based checkpoint blockade did not fully exhibit its antitumor function because the immunosuppressive tumor microenvironment plays a critical role in regulating the antitumor functions of NK cells. Thus, combining NK cell-based checkpoint blockade and targeting the vital specific components of the lung microenvironment would be a better solution for lung cancer therapy.

As the key immunosuppressive components of the lung tumor microenvironment, TGF-β and PGE2 have been widely studied. Targeting TGF-β or PGE2 in several studies has been shown to restore NK cell function in tumor therapy. However, the direct role of other components of the lung tumor microenvironment (glucose metabolism, hypoxia, and surfactant protein and lipids) is largely unknown. More basic research is needed to fully understand the relationship between these components and NK cell exhaustion. Notably, insights in the field of the lung tumor microenvironment and NK cell exhaustion will allow and facilitate the development of rationally designed combination immunotherapeutic strategies for lung cancers.

## Author contributions

HZ: Writing – original draft. JW: Writing – review & editing. FL: Writing – review & editing.
