# Corrigendum: Modulation of natural killer cell exhaustion in the lungs: the key components from lung microenvironment and lung tumor microenvironment

**DOI:** 10.3389/fimmu.2024.1467723

**Published:** 2024-07-30

**Authors:** Hongxia Zhang, Jian Wang, Fengqi Li

**Affiliations:** ^1^ Institute of Health and Medicine, Hefei Comprehensive National Science Center, Hefei, Anhui, China; ^2^ Department of Neurology, The First Affiliated Hospital of University of Science and Technology of China (USTC), Division of Life Sciences and Medicine, University of Science and Technology of China, Hefei, Anhui, China

**Keywords:** natural killer cells, hypofunction, exhaustion, lung microenvironment, lung tumor microenvironment

**Additional Affiliation(s)**

In the published article, there was an error regarding the affiliations for Jian Wang. As well as having affiliations 1, they should also have *Institute of Health and Medicine, Hefei Comprehensive National Science Center, Hefei, Anhui, China*.

The authors apologize for this error and state that this does not change the scientific conclusions of the article in any way. The original article has been updated.

**Incorrect Author Name**

In the published article, an author name was incorrectly written as Fenggqi Li. The correct spelling is Fengqi Li.

In addition, the corresponding author name in the information of the Correspondence was incorrectly written as Fenggqi Li. The correct spelling is Fengqi Li.

The authors apologize for this error and state that this does not change the scientific conclusions of the article in any way. The original article has been updated.

**Incorrect Funding**

In the published article, there was an error in the Funding statement. The description of the Funding title is incorrect for the Research Start-up Funding (2022KYQD010) and Global Select Project (DJK-LX-2022005) of the Institute of Health and Medicine, Hefei Comprehensive National Science Center, and the grant number is missing for the Fundamental Research Funds for the Central Universities. The correct Funding statement appears below.

**FUNDING**

The author(s) declare financial support was received for the research, authorship, and/or publication of this article. This work was supported by the Institute of Health and Medicine, Hefei Comprehensive National Science Center (2022KYQD010, DJK-LX-2022005), the R&D Program of Guangzhou Laboratory (SRPG22-006), the Strategic Priority Research Program of the Chinese Academy of Sciences (XDB0490000), the National Key Research and Development Program of China (2022YFC2304102), the Natural Science Foundation of China (82271827), and the Fundamental Research Funds for the Central Universities (WK3520000016).

The authors apologize for this error and state that this does not change the scientific conclusions of the article in any way. The original article has been updated.

